# Beyond strength: dietary nitrate augments self-selected functional mobility after resistance training in middle-aged adults

**DOI:** 10.1007/s11357-025-02048-z

**Published:** 2025-12-18

**Authors:** Brandon M. Peoples, Mason C. McIntosh, Kenneth D. Harrison, Bria R. Smith, Keven G. Santamaria Guzman, Silvia E. Campos-Vargas, Damaris C. Cifuentes, Breanna J. Mueller, Andreas N. Kavazis, Darren T. Beck, Michael D. Roberts, Jaimie A. Roper

**Affiliations:** 1 School of Kinesiology, Auburn University, 301 Wire Road, Office, Auburn, AL 36849 USA; 2 Edward Via College of Osteopathic Medicine, Auburn, AL USA

**Keywords:** Aging, Beetroot juice, Dietary nitrates, Gait, Functional mobility

## Abstract

This study aimed to assess whether dietary nitrate supplementation with resistance training (RT) augments gait performance and functional mobility in inactive middle-aged and older adults. We hypothesized that combining nitrate-rich beetroot juice (BRJ) with RT would yield improvements in gait and functional mobility compared to RT with nitrate-depleted beetroot juice placebo (PLA) supplementation. In this 12-week, randomized, double-blind, placebo-controlled pilot trial, 28 healthy, inactive adults (56 ± 7 years) were assigned to either a BRJ (~ 12.8 mmol nitrate/day) or PLA group while completing a supervised, full-body RT program (2×/week). Pre- and post-intervention assessments included the instrumented stand and walk (iSAW), 6-min walk test (i6MWT), and timed up and go (iTUG) tests. Supplement adherence, training compliance, and volume were equivalent between groups. ANCOVA analyses controlling baseline values revealed significant between-group differences favoring the BRJ group for gait speed (adjusted means: 1.38 vs 1.27 m/s, *p* = .010), stride length (*p* < .001), and iTUG duration (*p* < .05). Within-group analyses confirmed that the BRJ group experienced significant improvements in gait speed (*p* < 0.001) and iTUG duration (*p* < 0.05), while the PLA group showed no functional changes, indicating that 12 weeks of RT alone was insufficient to improve functional mobility in our sample despite equivalent training responses (*p* > .05). In our middle-aged sample, supplementing dietary nitrate alongside RT led to clinically meaningful improvements in functional mobility during self-selected conditions, exceeding the gains from 12 weeks of twice-weekly RT alone. Preliminary descriptive analyses revealed sex-specific group-level differences that might suggest emerging trends. Although no inferential statistics were conducted, the patterns provide potential directions for future research investigating the effects of RT in middle-aged adults.

**Graphical Abstract:**

**Figure:**
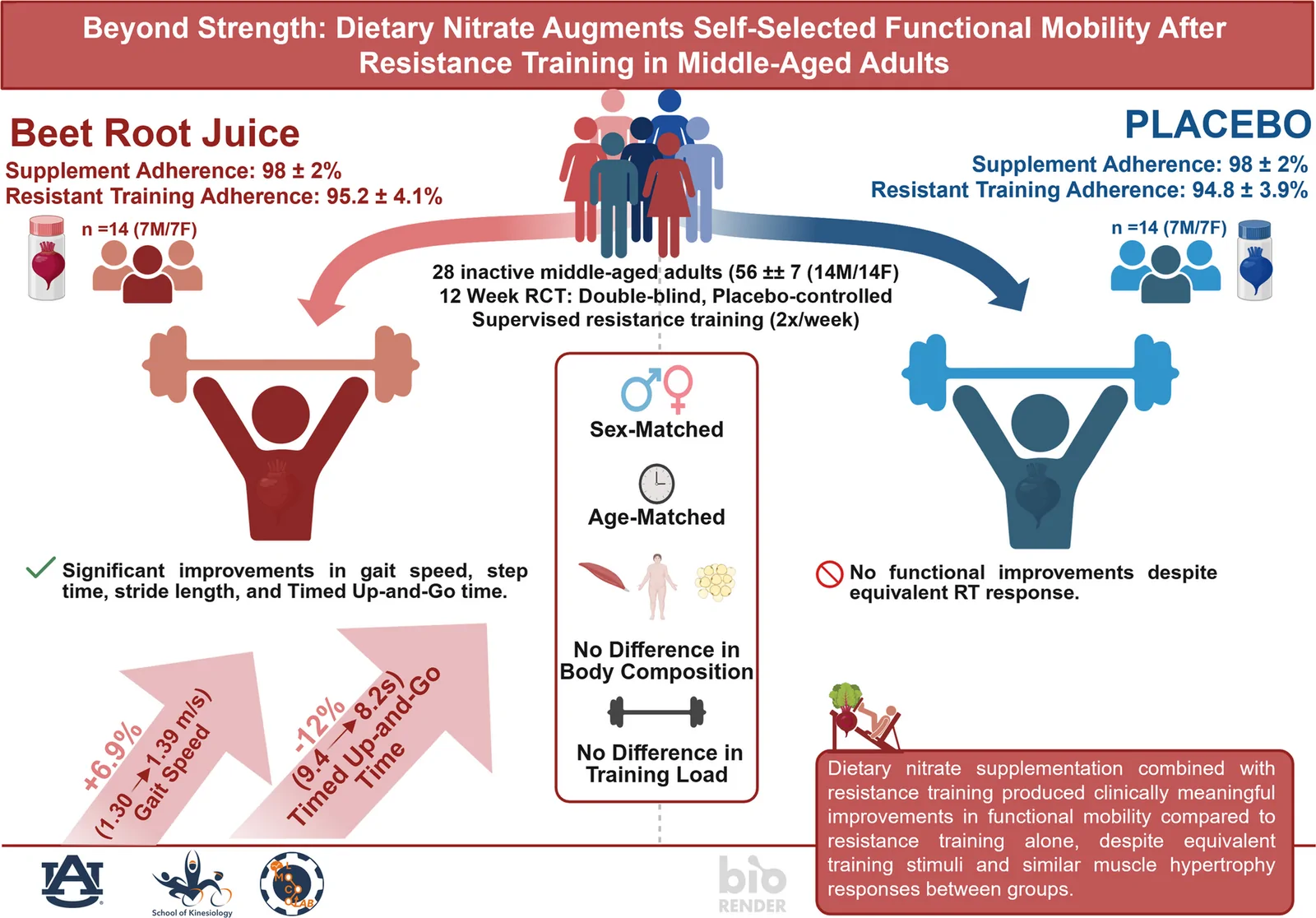

## Introduction

Mobility decline happens earlier than most realize, with functional deterioration (i.e., declines in gait speed) often appearing well before old age [1, 2]. While most gait and mobility research has focused on the differences between younger and older adults, the transitional period of middle adulthood remains largely overlooked [3, 4]. Extensive evidence shows that older adults walk more slowly [5–8], take shorter steps, spend more time in double support, and experience overall declines in functional mobility compared to their younger counterparts [6]. Moreover, physically active older adults often demonstrate distinct gait patterns compared to their less active peers [9], underscoring the influence of lifestyle on mobility. Yet, little is known about when these changes begin or whether interventions in middle adulthood can effectively improve gait and mobility during this stage of life. Middle adulthood, the period between early adulthood (ages 18–34) and late adulthood (65+), may represent a pivotal epoch for detecting and improving early signs of decline [3]. Therefore, investigating gait and mobility in middle adulthood may advance our understanding of how mobility declines with age and help identify the most effective preventative strategies.

Maintaining proper muscle function is key to ensuring adequate mobility, especially since sarcopenia often begins in early middle adulthood [10]. Two promising, non-pharmacological strategies for mitigating this decline are resistance training (RT) and dietary nitrate (NO_3_-) supplementation. RT is well established as a primary countermeasure against age-related declines in muscle mass and strength [11, 12]. Furthermore, RT has been shown to improve gait speed, timed-up-and-go performance, and physical function in older adults [13–15]. However, the findings on the effect of RT on mobility in middle-aged adults are relatively sparse. Meanwhile, dietary NO_3_-, abundant in vegetables such as beetroot and leafy greens, is proposed to improve endothelial function, enhance muscle contractility, and metabolic efficiency via nitric oxide pathways [16, 17]. The proposed mechanism involves the oral reduction of NO_3-_ to nitrite, which is subsequently converted to nitric oxide [18, 19]. This key signaling molecule can augment vasodilation and improve the efficiency of muscle contraction. While the potential ergogenic effects of NO_3_- have been explored in older adults, with some studies suggesting benefits such as increased fatigue resistance and improved functional mobility, the overall evidence remains equivocal [20–22]. Furthermore, a notable gap exists in research on the combined effect of RT and NO_3_- in middle-aged adults, underscoring the need for investigation into this combined approach.

Therefore, the primary purpose of this pilot and feasibility study was to (1) determine the feasibility, safety, and tolerability of a 12-week combined RT and BRJ supplementation protocol in inactive middle-aged adults, and (2) gather preliminary data on the intervention’s effect on functional mobility. The pre-specified primary functional outcome for this pilot investigation was self-selected gait speed. We hypothesized that combining RT with BRJ supplementation would yield superior improvements in our primary outcome compared to RT with placebo. Our primary hypothesis posited that combining RT with BRJ supplementation would yield superior improvements in gait and mobility metrics compared to RT alone. We anticipated that the combination of RT and BRJ would improve gait speed during self-selected and fast walking. Furthermore, we hypothesized that there would be marked improvements in gait characteristics (e.g., stride length, step time, and stride duration) and functional mobility, as measured by the timed-up-and-go test [23]. Within-group comparisons were conducted to determine whether RT intervention-related improvements occurred in each condition, although these tests were not used to evaluate the primary hypothesis. An exploratory descriptive analysis of sex-based differences within each group is presented. These hypotheses are supported by preliminary evidence indicating that BRJ supplementation and exercise improve functional connectivity within motor networks in older adults [24]. This manuscript reports on the functional mobility domain of a pilot and feasibility trial study designed to investigate the effects of BRJ and RT on distinct physiological systems. The primary outcome for this specific domain of the trial was self-selected gait speed, chosen for its strong predictive value for future health and clinical relevance [25–27].

## Methods

### Ethical approval

This study protocol was approved by the Auburn University Institutional Review Board (Protocol #24–863 MR 2405) and adhered to the guidelines set forth in the latest version of the Declaration of Helsinki. All procedures followed were in accordance with the institution’s guidelines. All participants provided written informed consent before participation.

### Participants

**Fig. 1:**
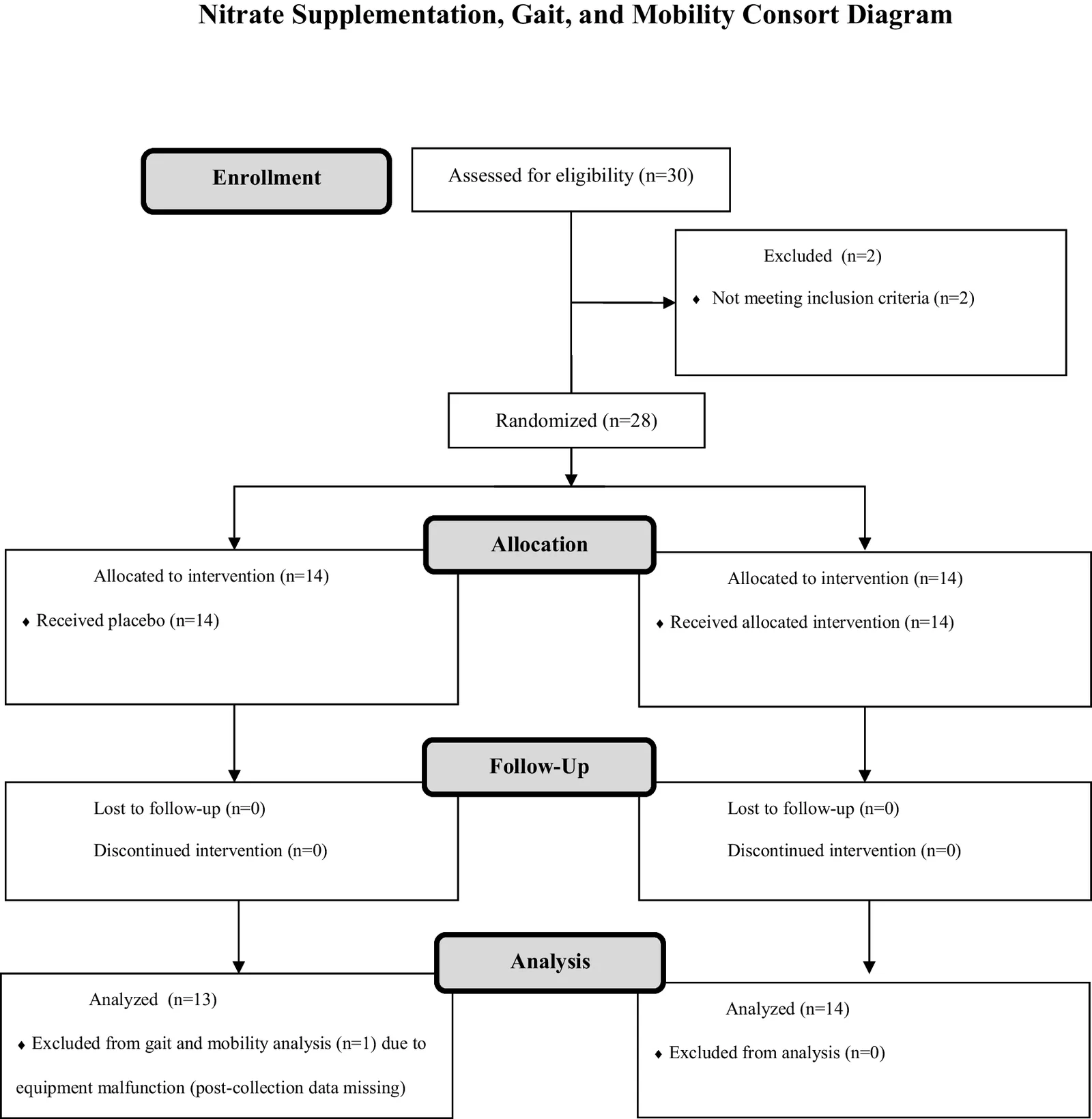
CONSORT flow diagram depicting the progression of participants throughout the study

Thirty inactive, community-dwelling middle-aged adults between the ages of 40 and 70 were recruited from the local community [28]. The inclusion criteria for study participation required participants to have exercised less than once a week for at least 1 year prior to recruitment. To control for vascular fluctuations associated with the menstrual cycle, only postmenopausal women were included [29, 30]. Exclusion criteria included (1) consumption of NO_3-_ rich supplements (e.g., beetroot juice), l-arginine, l-citrulline, or creatine-containing supplements within one month of the study’s start; (2) self-reported blood clotting disorders that would prevent them from undergoing skeletal muscle biopsies; (3) allergies to the study supplement or lidocaine; (4) any cardiometabolic conditions or orthopedic injuries that would preclude safe participation. These include hernias that have not been surgically corrected, diabetes, hypertension, unstable angina, severe osteoporosis, or any neurological conditions such as Parkinson’s disease. All participants were informed of the study procedures, risks, and benefits before providing written informed consent. After enrollment, participants were instructed to avoid alcohol-based mouthwash for the duration of the study to preserve NO_3-_ metabolism in the oral microbiota [31, 32]. Of the initial 30 recruits, 28 participants (14 M/14 F) enrolled and completed the study (see the CONSORT diagram in Fig. 1). Throughout the RT intervention, participants were monitored by certified training staff for any adverse events, defined as any musculoskeletal injuries related to the training or any self-reported negative reactions to the supplementation.

### Experimental design

**Fig. 2:**
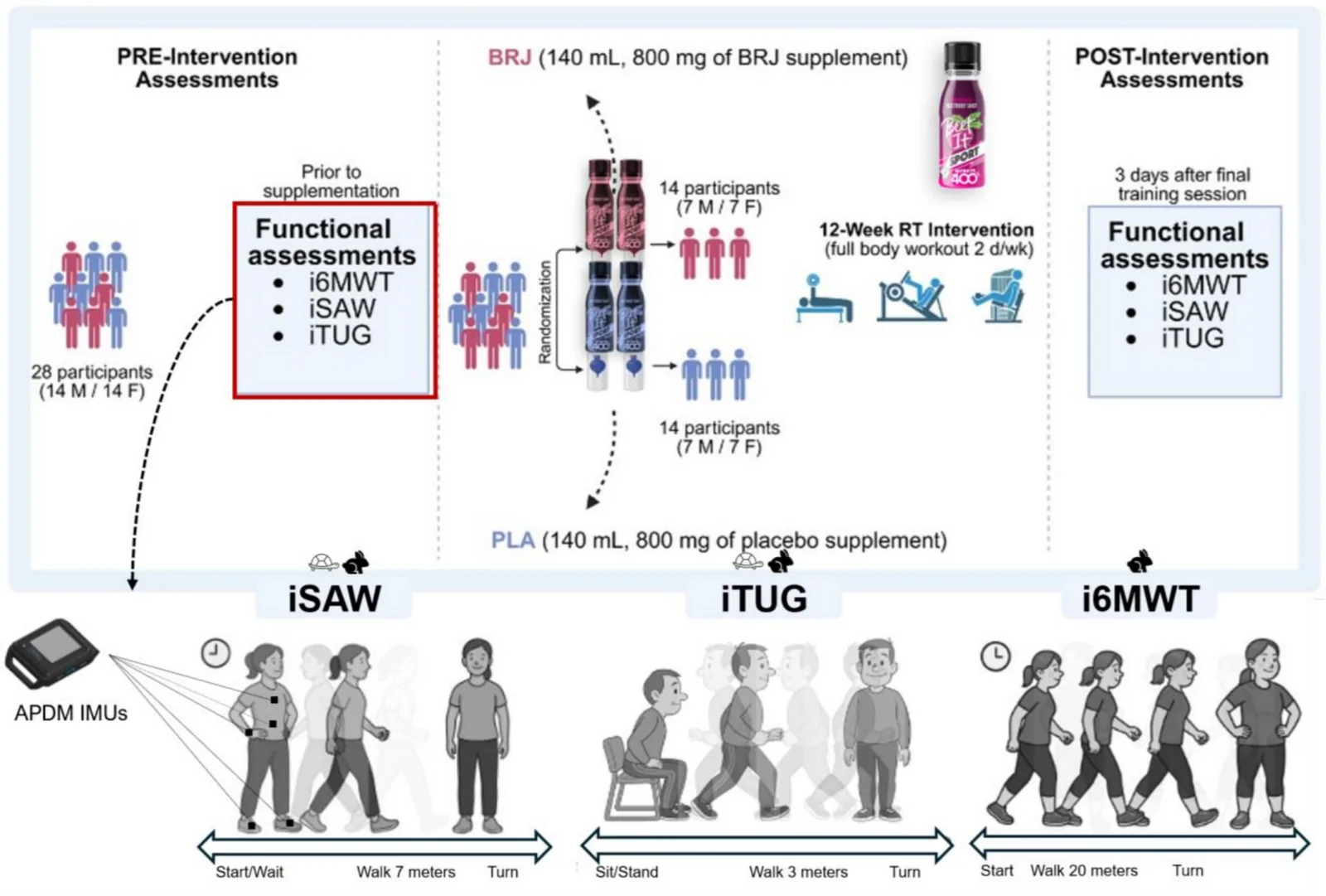
Overview of the study design, timeline, and assessments. Study participants underwent a series of pre-intervention functional assessments. Following these assessments, participants were randomized to receive either a nitrate-rich beetroot juice supplement (BRJ) or a nitrate-depleted placebo (PLA) daily for 12 weeks. Both groups concurrently participated in a supervised, full-body resistance training (RT) program twice per week. All assessments were repeated following the 12-week intervention. The bottom panel provides a visual breakdown of the key instrumented functional mobility tests: the instrumented Stand and Walk (iSAW), the instrumented Timed-Up-and-Go (iTUG), and the instrumented 6-Minute Walk Test (i6MWT). The tortoise icon indicates that the assessment was performed at a self-selected pace, while the rabbit icon indicates that the assessment was performed at a fast pace. IMUs, Inertial

This study employed a randomized, double-blind, placebo-controlled experimental design, as illustrated in Fig. 2. Pre-intervention (PRE) and post-intervention (POST) assessments were conducted to evaluate the effects of a 12-week full-body RT intervention combined with NO_3_- supplementation, as previously reported [33]. During PRE testing, participants arrived at the laboratory in a fasted state (≥ 4 h). Instrumented gait and mobility assessments were conducted before strength testing and are detailed below. Participants then completed a 12-week, twice-weekly RT intervention followed by post-intervention (POST) testing. A detailed description of these study procedures is reported elsewhere [33, 34].

### Dietary supplementation

Following PRE testing and before training, participants were block randomized to either a NO_3_^-^-depleted BRJ placebo (PLA) or a NO_3_^-^-rich BRJ (BRJ) group according to age, sex, and DXA lean tissue mass; both supplements were Beet It Sport shots (James White Drinks Ltd, UL; 70 mL, Ipswich, UK). Beet It Sport contains 98% concentrated beetroot juice and 2% concentrated lemon juice. Beginning on the first day of the exercise intervention, participants were instructed to consume two shots per day of their assigned supplement. NO_3_^-^ dosage provided from the 140 mL/day included ~ 0.08 mmol NO_3_^-^ for the PLA group and ~ 12.8 mmol NO_3_^-^for the BRJ group. Participants received weekly supplements in identical, indistinguishable bottles from a research assistant who was not involved in data collection, statistical analysis, or participant interaction, ensuring double blinding. The nitrate-depleted placebo was identical in taste, appearance, and content of other bioactive compounds (e.g., betalains and polyphenols) to the BRJ, allowing for the specific effect of NO_3_- to be isolated. Participants were instructed to consume their supplement dosage 2.5 h before each training session and in the morning on non-training days. Previous studies suggest that NO_3_- should be consumed 2–3 h before exercise to maximize its benefits on exercise performance [32, 35]. Compliance with supplementation was confirmed through self-reported data collected from monthly questionnaires.

### Resistance training intervention

Participants engaged in a 12-week, full-body RT intervention twice weekly on non-consecutive days. All sessions were supervised by a certified strength and conditioning specialist. The program was structured to emphasize progressive overload. For weeks 1–4, participants performed 3 sets of 12–15 repetitions. The training intervention progressed from 3 sets of 10–12 repetitions for weeks 5–8 to 3 sets of 8–10 repetitions for weeks 9–12. Training loads were adjusted based on a 0–10 repetition in reserve scale to ensure participants maintain a relatively high exertion for their training loads. RIR is a precise tool for adjusting RT loads for individual capability, regardless of experience [36, 37]. RT training loads remained the same if participants reported an RIR of 2 or higher; however, loads were reduced if participants reported an RIR of 0 or were unable to maintain the targeted repetition range.

### Training compliance and load monitoring

Training attendance and progression were systematically monitored throughout the 12-week intervention. Both groups demonstrated excellent and comparable training adherence, with BRJ participants attending 95.2 ± 4.1% of sessions and PLA participants attending 94.8 ± 3.9% of sessions. Individual training loads were tracked, with no significant differences in total training volume-load between groups, confirming equivalent exercise stimulus across conditions [33].

### Gait and functional mobility assessments

Gait and functional assessments were instrumentally evaluated using the APDM Mobility Lab system with Opal inertial measurement units (IMUs) (APDM, Inc., Portland, OR, USA) [38]. IMUs were placed on the following locations: (1) on the chest, centered over the upper part of the sternum; (2) on the top area of the lower back near the sacrum (just below the lower end of the spine); (3) on the middle of the back side of the wrist bones; and 4) on the top surface of each foot, centered over the middle and outer bones [39]. All assessments were performed in a quiet hallway with distance labeled and marked with landmarks.

### Instrumented Stand and Walk (iSAW)

Participants stood upright with their feet shoulder-width apart, hands on their hips, and eyes forward for a 30-s quiet balance. After maintaining the quiet balance, participants walked 7 m to a marked landmark, turned around, and then walked 7 m back to their starting position, facing forward [40]. The iSAW was performed for a total of four trials at two different speeds, including once at a self-selected normal walking speed and once at their fastest possible safe walking speed. Both trials were averaged for all gait spatiotemporal outcomes for each walking speed condition.

### Instrumented Timed Up and Go (iTUG)

Participants started sitting with arms crossed and hands resting on their shoulders. Researchers then instructed them to stand up, emphasizing that they should use their hands only when necessary. Participants walked 3 m, turned, walked back, and then sat back down. The iTUG was also performed for a total of four trials at two different speeds, including once at a self-selected normal walking speed and once at their fastest safe walking speed. Both trials were averaged, and the overall duration and subphase durations (sit-to-stand, stand-to-sit, and turning) were analyzed.

### Instrumented 6-min Walk Test (i6MWT)

Participants started standing with their hands resting against their sides. They were then instructed to walk at their fastest, yet safest, walking speed back and forth along a quiet 20-m hallway for 6 min, covering as much distance as possible. The walking course was marked at every meter. Participants were permitted to take breaks, although no participant required one. The overall distance was analyzed by totaling the number of laps completed and the marked meter distance, as measured by two research team members, who stopped participants at the end of the test.

### Primary and secondary outcomes

Given the clinical relevance and known minimal clinically important differences, the self-selected gait speed during the iSAW was chosen as the main outcome measure. Secondary outcomes included stride length, step time, stride time, iTUG duration, and i6MWT distance. All secondary analyses were conducted on self-selected and fast-paced gait walking speeds. This hierarchy was established to control type I error across multiple comparisons.

### Statistical analysis

All statistical analyses were performed blinded using JMP 18 Pro (SAS Institute Inc., Cary, NC, USA) and JASP stats (JASP Team, Version 0.19.3). Data were visualized using the *ggpubr* package in RStudio version 2024.12.1+563 (Posit Software, PBC, Boston, MA) and JMP 18 Pro [41]. All data were tested for normality using the Shapiro–Wilk test and visually inspected using Q-Q plots. Independent t-tests were used to assess relative changes in demographics, body composition, adherence, and lower extremity strength between the BRJ and PLA groups. Model assumptions, including linearity, homoscedasticity, and residual independence, were validated using diagnostic plots. As this was a pilot study, an a priori power analysis was performed using G*Power (version 3.1.9.7) to determine the necessary sample size to detect a large effect in our pre-specified primary functional outcome (self-selected gait speed) in an ANCOVA model with one covariate (PRE gait speed) and one fixed factor (group: BRJ vs. placebo). The anticipated effect size (*f* = 0.6) was based on a systematic review and meta-analysis by Hortobágyi et al. (2015), which reported a between-group effect size of 1.15 for RT interventions on habitual gait speed in healthy older adults [13]. Assuming α = 0.05 and power = 0.80, the required sample size was 24 participants (12 per group). The analysis was intended to ensure our preliminary study was sufficiently sensitive to this main effect, which would inform the design of a future, larger-scale trial.

A single ANCOVA was performed for the primary outcome (self-selected gait speed) with *α* = 0.05, using post-intervention gait speed as the dependent variable, group as the independent variable, and pre-intervention gait speed as the covariate. The assumptions for ANCOVA were checked for each variable before analysis, including the absence of significant outliers, the normal distribution of residuals for each group, homogeneity of variances (Levene’s test), a linear relationship between the covariate and the dependent variable, homoscedasticity, and homogeneity of regression slopes.

Secondary outcomes were analyzed using separate ANCOVAs with the same covariate approach. To control the family-wise error rate, the Benjamini–Hochberg false discovery rate (FDR) procedure was applied across all secondary outcomes during self-selected and fast walking conditions using a *q*-value of 0.05.

To evaluate the effect of RT on each group, within-group comparisons were conducted using paired-samples *t*-tests to assess the PRE-POST effects of the RT intervention. As part of the exploratory analysis, sex mean differences for each group were examined descriptively without formal statistical testing; these comparisons were intended to identify potential trends and inform future hypothesis generation.

## Results

### Baseline group characteristics

The 28 participants who completed the study were successfully randomized into age- and sex-matched BRJ (*n* = 14, 7 M) and PLA (*n* = 14, 7 M) groups. At baseline, there were no statistically significant differences in age, height, body mass, or body mass index. Additionally, body composition measures, such as the fat mass-to-fat-free mass ratio (FM/FFM), total lean mass, and total fat mass, were similar between the groups. As previously reported, supplementation adherence was outstanding, with BRJ reporting 98 ± 2% and PLA participants 99 ± 2% compliance, showing no statistical difference (*p* > 0.05) [33]. We also previously reported no differences in estimated one-repetition max strength via the 5-RM hex bar deadlift, training volume, or knee extensor strength assessed via isokinetic dynamometry (p > 0.05) [33, 34]. Collectively, these findings indicate that the groups were matched not only for age and sex but also balanced in terms of body composition, training load, and strength. Table 1 below displays the PRE intervention characteristics of each group. The RT intervention was well tolerated. Participants in either group reported no adverse events or musculoskeletal injuries related to the training or supplementation during the 12-week intervention.

**Table 1:** Pre-intervention/post-intervention group characteristics

Variable	BRJ (*n* = 14)		Rel change (%Δ)	PLA (*n* = 14)		Rel change (%Δ)	***p***
	PRE	POST		PRE	POST		
Age (years)	56 ± 6	-	-	56 ± 7	-	-	-
Sex	7 M/7 F	-	-	7 M/7 F	-	-	-
Height (m)	174 ± 8	-	-	172 ± 13	-	-	-
Systolic BP	125 ± 13	130 ± 17	4.3 ± 11.5	128 ± 15	127 ± 19	−0.8 ± 10.2	0.25
Diastolic BP	79 ± 6	81 ± 8	2.2 ± 9.7	80 ± 10	81 ± 12	3.0 ± 17.2	0.88*
Total mass (kg)	86.7 ± 17.3	87.3 ± 16.2	0.9 ± 2.1	84.6 ± 23.7	85.0 ± 23.9	0.5 ± 3.7	0.73
BMI (kg/m^2^)	29.4 ± 5.6	28.9 ± 4.8	− 1.4 ± 2.9	28.9 ± 5.1	28.3 ± 5.1	− 1.9 ± 3.8	0.74
FM/FFM	0.60 ± 0.2	0.60 ± 0.2	− 1.0 ± 5.4	0.63 ± 0.18	84.6 ± 23.7	− 1.5 ± 7.5	0.85
Total lean mass (kg)	51.4 ± 9.1	52.1 ± 8.6	1.4 ± 2.6	50.4 ± 14.0	51.0 ± 14.3	1.2 ± 2.3	0.82
Total fat mass (kg)	32.4 ± 11.3	32.4 ± 11.1	0.2 ± 4.5	31.4 ± 12.2	31.2 ± 12.6	− 0.4 ± 7.8	0.79
Total training volume							

*BMI*, body mass index; *FM/FFM*, fat mass to fat-free mass ratio, *indicates normality was violated, and the Mann–Whitney *U* test was performed instead

### Primary outcome

**Fig. 3:**
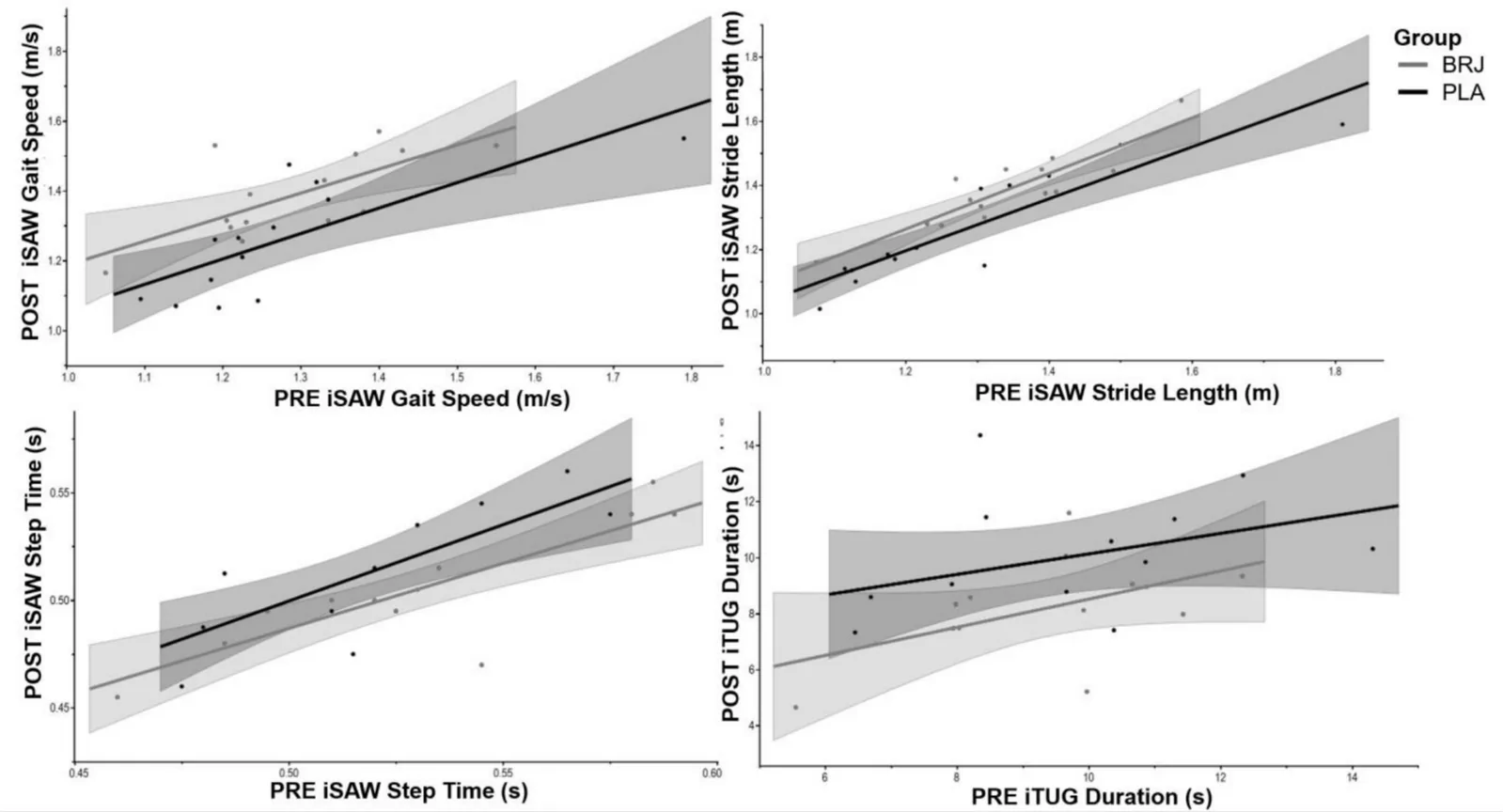
ANCOVA scatterplot for pre- and post-RT gait and mobility measures. Figure 2 shows scatter plots of individual outcomes for BRJ and PLA groups, illustrating the differences between pre- and post-intervention measures in gait speed, stride length, step time, and iTUG duration. Lines of best fit and 95% confidence intervals indicate stronger post-test improvements among BRJ participants across multiple parameters. Panel **a** displays pre- to post-intervention gait speed (m/s); **b** shows pre- to post-intervention stride length (m); **c** depicts the relationship between pre-intervention iTUG duration (s) and post-intervention step time (s); and **d** presents pre- to post-intervention iTUG duration (s). Each panel emphasizes potential intervention effects and baseline function relationships, with visual differentiation between BRJ and PLA regression lines

For our primary outcome, self-selected gait speed, ANCOVA revealed a significant difference between the groups after controlling for baseline values (*F*(1, 24) = 7.92, *p* = 0.01, ηp^2^ = 0.25), as shown in Fig. 3. The BRJ group showed significantly faster gait speeds (adjusted *M* = 1.38 m/s, SE = 0.03) compared with the placebo (PLA) group (adjusted *M* = 1.27 m/s, SE = 0.03) (Fig. 3a).

### Secondary outcomes—self-selected walking

After applying a false discovery rate (FDR) correction across all secondary outcomes, significant group differences remained. The BRJ group demonstrated a significant improvement in stride length compared to the PLA group (Fig. 3b) (*F*(1,24) = 8.10, *q* < 0.001, ηp^2^ = 0.25) and a shorter step time (*F*(1, 24) = 4.55, *q* < 0.05, ηp^2^ = 0.16) (Fig. 3c). Additionally, the BRJ group experienced a significantly greater improvement (i.e., faster time) in iTUG duration (*F*(1,23) = 5.41, *q* < 0.05, ηp^2^ = 0.19) (Fig. 3d). There was no significant difference in stride duration (*F*(1, 24) = 3.46, *p* = 0.08, FDR-adjusted *q* > 0.05).

### Secondary outcomes—fast-paced walking

No significant group effects were observed for gait speed, step time, stride length, or stride time. Additionally, there was no statistically significant difference in iTUG duration or walking capacity, as assessed by the 6MWT distance at fast walking speed (all *q* > 0.10).

### Within-group changes in response to resistance training

**Fig. 4:**
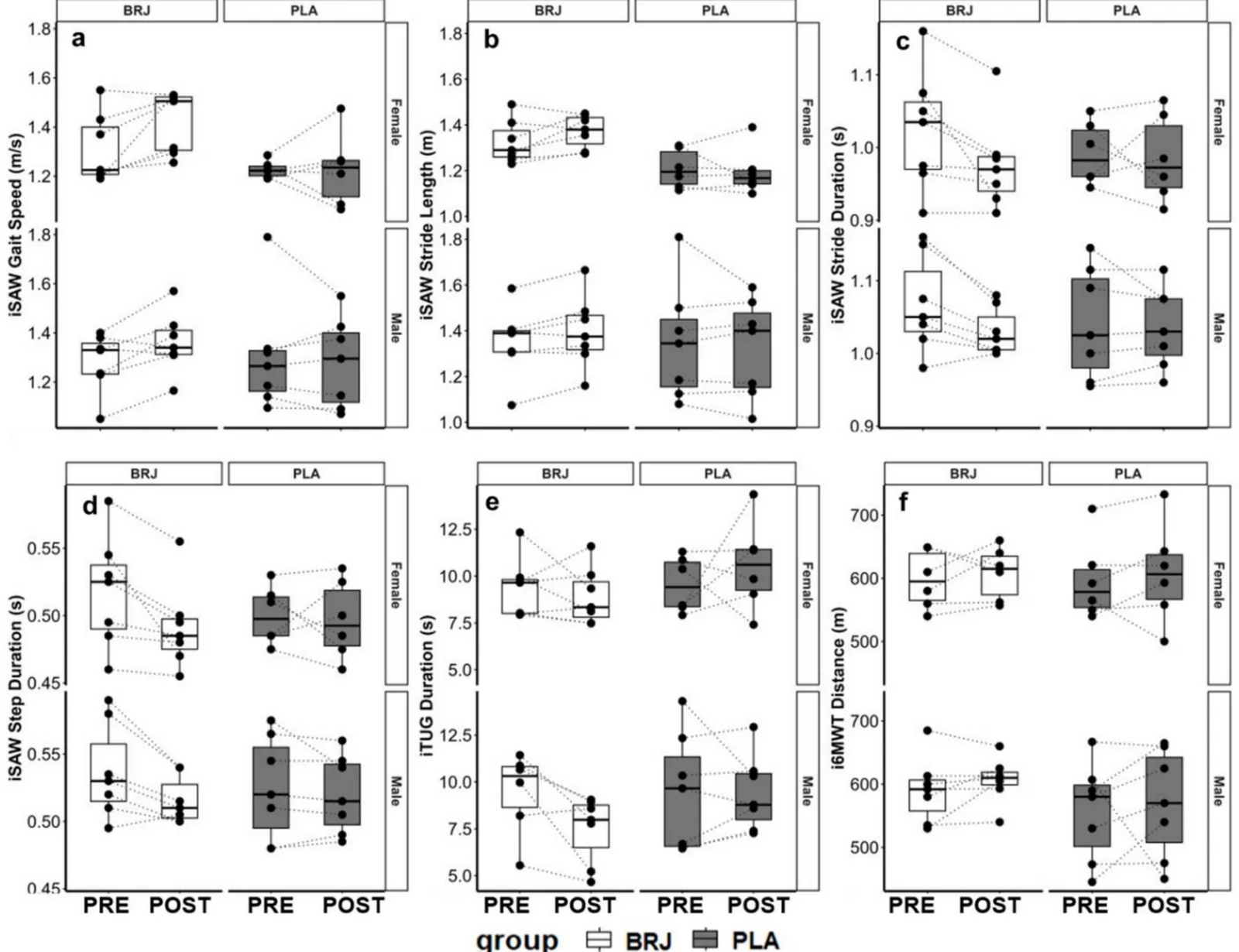
Pre–post changes in gait and mobility metrics following 12 weeks of resistance training with beetroot juice (BRJ) or placebo (PLA) supplementation. Panels **a**–**d** display gait metrics during the instrumented Stand and Walk test (iSAW): **a** gait speed (m/s), **b** stride length (m), **c** stride duration (s), and **d** step duration (s). Panel **e** shows instrumented Timed Up and Go (iTUG) duration (s), and panel **f** shows distance covered during the instrumented 6-Minute Walk Test (i6MWT) in meters. The panels have been facet-wrapped by group and sex. Box plots represent median values with interquartile ranges; individual data points are connected by lines to illustrate within-subject changes from PRE to POST. BRJ participants demonstrated consistent improvements in gait speed, stride length, and temporal parameters (stride duration and step time), as well as reductions in TUG duration and minimal increases in 6MWT distance. In contrast, PLA participants showed minimal or inconsistent changes across most metrics. These results suggest that BRJ supplementation may enhance mobility outcomes when paired with resistance training, particularly in habitual gait and transitional tasks

To provide context for these between-group differences and the effects of RT, within-group changes from pre- to post-intervention were assessed. The BRJ group demonstrated significant improvements in self-selected gait speed, increasing from 1.30 ± 0.13 m/s to 1.39 ± 0.12 m/s (*p* < 0.001, Cohen’s *d* = − 0.98), seen in Fig. 4a. This was accompanied by a significant increase in stride length (*p* < 0.01, Cohen’s *d* = − 0.80) (Fig. 4b) and a corresponding quicker step (*p* < 0.001, Cohen’s *d* = 1.12) (Fig. 4c) and gait cycle measure via stride duration (*p* < 0.001, Cohen’s *d* = 1.01) (Fig. 4d). The BRJ group also showed a significant decrease in iTUG duration, from 9.4 ± 1.8 s to 8.2 ± 1.8 s (*p* < 0.05, Cohen’s *d* = 0.65) (Fig. 4e). Participants in both groups walked further during the i6WT, but these increases were not statistically significant (*p* > 0.05) (Fig. 4f). In contrast, the PLA group showed no statistically significant improvements in any of the measured functional outcomes during either self-selected or fast-paced trials (all *p* > 0.05). These within-group findings reveal that RT combined with NO_3_- supplementation produced significant improvements in functional outcomes (Cohen’s *d* = 0.65–1.12). In contrast, the 12-week 2-day RT intervention alone produced no measurable functional benefits, despite equivalent gains in strength and muscle mass, as previously reported.

### Exploratory sex differences

Changes in gait speed, stride length, step time, and stride time varied across sex, condition, and group in the iSAW (Table 2). The BRJ group demonstrated consistent improvements in gait speed across both sexes, with females showing greater gains under fast conditions and males under self-selected conditions. Stride length increased during self-selected walking in both sexes but decreased slightly during fast walking. Step time and stride time decreased across all BRJ participants. In contrast, the PLA group showed minimal or inconsistent changes, with females exhibiting slight improvements in stride length only during fast walking and males showing no change or minor gains.

**Table 2:** Effects of resistance training and dietary nitrate supplementation on mobility outcomes across sex and walking condition in middle-aged adults

	Sex	Condition	Group	Mean difference	Difference direction
**Distance (m)**					
**i6MWT**	Female	**FAST**	BRJ	10.20	↑ Increase in distance
	Female		PLA	11.50	↑ Increase in distance
	Male		BRJ	15.90	↑ Increase in distance
	Male		PLA	13.30	↑ Increase in distance
**Gait Speed (m/s)**					
	Female	**SSW**	BRJ	0.11	↑ Increase in gait speed
	Female		PLA	0.00	↔ No change
	Male		BRJ	0.08	↑ Increase in gait speed
	Male		PLA	− 0.03	↓ Decrease in gait speed
**iSAW**	Female	**FAST**	BRJ	0.07	↑ Increase in gait speed
	Female		PLA	0.03	↑ Increase in gait speed
	Male		BRJ	0.02	↑ Increase in gait speed
	Male		PLA	0.01	↑ Increase in gait speed
**Stride Length (m)**					
**iSAW**	Female	**SSW**	BRJ	0.05	↑ Increase in stride length
	Female		PLA	− 0.01	↓ Decrease in stride length
	Male		BRJ	0.04	↑ Increase in stride length
	Male		PLA	− 0.03	↓ Decrease in stride length
	Female	**FAST**	BRJ	0.00	↔ No change
	Female		PLA	0.01	↑ Increase in stride length
	Male		BRJ	0.00	↔ No change
	Male		PLA	0.02	↑ Increase in stride length
**Step Time (s)**					
	Female	**SSW**	BRJ	− 0.03	↓ Decrease in step time
	Female		PLA	0.00	↔ No change
	Male		BRJ	− 0.02	↓ Decrease in step time
**iSAW**	Male		PLA	− 0.01	↓ Decrease in step time
	Female	**FAST**	BRJ	− 0.02	↓ Decrease in step time
	Female		PLA	− 0.01	↓ Decrease in step time
	Male		BRJ	− 0.01	↓ Decrease in step time
	Male		PLA	0.00	↑ Increase in step time
**Stride time (s)**					
	Female	**SSW**	BRJ	− 0.05	↓ Decrease in stride time
	Female		PLA	− 0.01	↓ Decrease in stride time
	Male		BRJ	− 0.04	↓ Decrease in stride time
**iSAW**	Male		PLA	− 0.01	↓ Decrease in stride time
	Female	**FAST**	BRJ	− 0.03	↓ Decrease in stride time
	Female		PLA	− 0.01	↓ Decrease in stride time
	Male		BRJ	− 0.02	↓ Decrease in stride time
	Male		PLA	0.00	↔ No change
**iTUG Duration (s)**					
**iTUG**	Female	**SSW**	BRJ	− 0.43	↓ Decrease the time
	Female		PLA	0.79	↑ Increase the time
	Male		BRJ	− 2.04	↓ Decrease the time
	Male		PLA	− 0.22	↓ Decrease the time
	Female	**FAST**	BRJ	− 1.22	↓ Decrease the time
	Female		PLA	0.15	↑ Increase the time
	Male		BRJ	− 0.04	↓ Decrease the time
	Male		PLA	− 0.87	↓ Decrease the time

*i6MWT*, instrumented 6-min walking test; *iSAW*, instrumented Stand and Walk test; *iTUG*, instrumented Timed Up and Go; *SSW*, self-selected walking speed; *FAST*, fast walking speed

In the i6MWT, both male and female participants demonstrated increased walking distance across both groups under fast conditions, with males in the BRJ group showing the largest improvement (15.9 m). In the iTUG, females in the PLA group showed increased completion time at both walking speeds. In contrast, all other groups showed reductions in iTUG duration, with the most substantial improvements observed in males from the BRJ group under self-selected conditions (− 2.04 s).

## Discussion

The purpose of our study was to examine whether dietary NO₃⁻ supplementation with RT produced functional mobility improvements in previously inactive middle-aged adults. In partial agreement with our hypotheses, we report three main findings: (1) RT with daily supplementation of NO_3_^-^-rich BRJ produces significant improvements in self-selected gait performance and mobility compared to the NO_3_^-^-depleted PLA group, (2) NO_3_^-^supplementation with RT did not confer additional benefit to gait performance and functional mobility during fast-paced walking speed conditions, and (3) 12 weeks of a 2-day-per-week progressive RT program, without concurrent NO_3_- supplementation, did not yield meaningful improvements in gait and mobility outcomes in middle-aged adults in our study. Collectively, these results highlight the potential of NO_3_- supplementation with RT in enhancing the quality and efficiency of movement in daily contexts, particularly under conditions that mimic habitual walking patterns. This insight suggests a potential shift in the application of NO_3_^-^ supplementation for aging populations. Rather than targeting muscle hypertrophy or endurance, the focus may be better placed on improving movement economy to support activities necessary for autonomy and quality of life in the fifth decade and beyond.

### Dietary nitrate and resistance training augments clinically relevant functional mobility

Our study’s primary finding is that a 12-week, 2-day RT intervention combined with BRJ supplementation improved functional mobility and gait quality beyond that of RT alone. Participants in the BRJ exhibited clinically meaningful improvements in their self-selected gait speed and iTUG duration compared to those in the PLA group. The significance of these specific improvements is substantial, as gait speed is widely regarded as a “sixth functional vital sign” and a powerful predictor of future adverse health events [25–27], and the iTUG is a cornerstone assessment for evaluating fall risk and functional decline [23, 42–44].

The average 0.1 m/s improvement in self-selected gait speed observed in the BRJ group meets the established minimal clinically important difference [13]. Similarly, the BRJ group’s 1.2-s decrease in iTUG duration represents a meaningful detectable change in the ability to navigate one’s environment. For context, Smith et al. followed middle-aged and older adults through a 6-week exercise program and established that the minimum detectable change for an iTUG test was 0.77 s under single-task conditions [45]. Their findings suggest that changes of this magnitude represent meaningful improvements in mobility for active older adults. As our BRJ group’s 1.2-s improvement exceeds this threshold, it likely reflects a clinically relevant improvement in functional mobility.

### Selective improvements implicate central neural mechanisms

The pattern of improvement in self-selected functional mobility not only offers insight into the potential mechanisms behind these changes but also is reflected in both quantitative and qualitative measures. These improvements include significantly longer stride lengths and quicker step and gait cycles, indicating notable augmentations in gait mechanics. This pattern suggests that the observed benefits of BRJ combined with RT may have been mediated by enhancements in self-selected gait speed during the straight-line walking portion of the iTUG, which have significant implications for exercise and the aging brain. Previously, Petrie et al. demonstrated that BRJ supplementation, combined with aerobic exercise, improved somatomotor community consistency and produced brain networks that more closely resembled those of younger adults. Their finding suggests that BRJ facilitates neural plasticity beyond the effects of exercise alone [24]. However, there has been a paucity of research regarding this topic. Thus, follow-up studies investigating RT, dietary NO₃⁻, and neuroimaging are needed to support this hypothesis.

A second significant observation underpinning this proposed mechanism is the differential effect manifested between the two walking speed conditions, with clinically relevant improvement exclusively during habitual, self-selected tasks in the BRJ. No statistically significant differences were detected between or within groups during maximal-speed assessments (i.e., the “fast” iSAW, iTUG, and i6MWT). This discrepancy may reflect the fact that self-selected gait is highly regulated by central neural processes, while maximal-speed tasks are more constrained by decreased cardiovascular capacity [46], peripheral sensory loss [47], or skeletal muscle morphology [48]. Although modest increases in fast gait speed were observed across both groups, these gains fell below the 0.12 m/s threshold reported in a meta-analysis by Hortobágyi et al. [13], suggesting that the BRJ + RT intervention may augment mobility within the bounds of habitual motor control rather than maximal exertion. Furthermore, differences in population characteristics (older vs. middle-aged adults), intervention duration (~ 12 vs. ~ 15 weeks), and training frequency (2 days vs. 3 days) may further explain the divergence in our findings. Our findings align with those of Khodadad Kashi et al. [14], who reported that improvements in muscular strength do not necessarily translate to 6MWT performance, reinforcing the notion that peripheral factors may constrain maximal-speed mobility outcomes in older adults.

### Exploratory sex-specific responses in gait and mobility

While our study was not powered for sex-specific subgroup comparisons, exploratory descriptive statistics revealed unique responses to the RT intervention. Male participants in both groups demonstrated improvements in mobility, but the nature and magnitude of these gains differed. Those in the BRJ group showed the most substantial reductions in iTUG duration, suggesting improved dynamic balance, transitional speed, and neuromuscular coordination [43]. These improvements are particularly relevant for older males, who often retain some muscle mass and strength but may experience age-related declines in movement efficiency and postural control [49]. In contrast, males in the PLA group showed modest improvements in iTUG duration, but these gains were less consistent.

Conversely, middle-aged females in the PLA group exhibited the least favorable response to the twice-weekly RT intervention, with minimal or no improvements across most mobility outcomes. Unlike their BRJ counterparts, female participants in the PLA group showed negligible changes or even slight declines in these domains. For example, their gait speed remained unchanged, and their stride length decreased slightly under self-selected walking conditions. These observations suggest that twice-weekly RT alone, at the prescribed frequency and duration, may be insufficient to elicit measurable improvements in mobility for some middle-aged females [11]. Therefore, these findings underscore the importance of considering sex-specific responsiveness and the potential need for adjunctive strategies to optimize mobility outcomes in aging populations.

### Methodological considerations and population

An important consideration in this study is the use of functional assessments (iTUG, iSAW, i6MWT) that are traditionally associated with geriatric or clinically impaired populations. Our rationale for this choice was based on growing evidence that functional decline often begins in middle adulthood, yet may be too subtle to detect with standard clinical tests (e.g., a stopwatch TUG) in a relatively healthy cohort [45, 50]. The iSAW, iTUG, and i6MWT provide more insight into the subtle, subclinical changes in gait metrics—such as stride length and step time—that signal upcoming, clinically observable functional impairments [40, 45, 51]. This heightened sensitivity was necessary for identifying the effects observed within our study.

It remains plausible that these results would differ in an older, frailer population. For instance, the 12-week, twice-per-week RT program alone (our PLA group) may have been a sufficient stimulus to improve functional mobility in frail older adults [13, 14, 52], which was not the case in our middle-aged sample. Furthermore, a population with greater initial impairment (i.e., a lower baseline) may possess a larger “window” for improvement, and the absolute magnitude of the benefit from combined BRJ + RT could potentially be greater. In contrast, adults with a history of resistance training may have a smaller window for improving functional mobility, but they may experience greater muscular endurance during training [53]. Therefore, our findings should be interpreted as specific to this transitional middle-aged cohort.

### Strengths and limitations

This investigation possesses several methodological strengths that bolster the validity of our results. The randomized, double-blind, placebo-controlled design, coupled with rigorous maintenance of blinding throughout data collection and analysis, effectively minimizes potential biases that could otherwise explain the observed functional improvements. The supervised, progressive RT protocol ensured high adherence rates (> 95% in both groups), with comparable training volume and loads, thereby confirming that the functional differences observed were not attributable to variations in training regimens. Lastly, this is one of the handful of studies to investigate the effects of RT and functional mobility in middle-aged adults.

We acknowledge several limitations when interpreting these findings. A key limitation, inherent to this study’s nature as a pilot trial, is the modest sample size. The study was powered a priori to detect a large, clinically meaningful between-group difference in our primary outcome (gait speed), not to detect smaller, secondary within-group changes. Therefore, the lack of a statistically significant functional improvement in the PLA (RT-only) group should be interpreted with caution. It is possible that 12 weeks of RT did provide a benefit, but our study was underpowered to detect this within-group effect. The large effect sizes observed should be cautiously interpreted until confirmed in larger studies. A larger, fully powered RCT would be needed to definitively determine the independent effect of this RT program on mobility in this population. In this study, the influence of participants’ dietary intake was not controlled for, representing a significant confounding variable. Accounting for dietary habits, particularly the consumption of natural sources of dietary nitrate (NO_3_-), would have mitigated potential additive effects that could influence the study’s outcomes. The absence of systematic dietary nitrate monitoring constitutes a significant gap, as background nitrate intake exhibits considerable variability among individuals and may influence both tissue nitrate accumulation and functional outcomes. Plasma nitrate and nitrite concentrations were not measured during active supplementation, thereby precluding confirmation of adequate circulating levels necessary for effective tissue uptake. The implementation of a 24-h supplement washout period before post-intervention testing, while designed to isolate chronic training adaptations from acute nitrate effects, introduces complexity in interpreting our findings. This complexity is coupled with our pre-exercise dosing strategy. We hypothesized that chronically training in a state of high nitric oxide bioavailability (by dosing 2.5 h before exercise) would lead to superior adaptations [35]. However, if the supplement were consumed right before the session, it is unlikely any benefit would be seen, as this is insufficient time for the pharmacokinetic conversion of NO_3_- to NO_2_-. Conversely, consuming the supplement immediately after exercise would test a different hypothesis, one related to recovery rather than performance augmentation during the bout. Additionally, our study population comprised relatively healthy, sedentary middle-aged adults; however, this sample limits the generalizability of the findings to frail older populations. Moreover, our study design included only resistance-trained groups (BRJ + RT and PLA + RT) without a non-exercise “nitrate-only” group. While this design allows us to conclude that nitrate supplementation augmented the effects of RT on gait and functional mobility, we cannot definitively determine the individual effects of nitrate as a synergistic interaction. Future studies with a nitrate-only group would be necessary to clarify whether this combination is effective.

## Conclusions

In conclusion, the results of this double-blind, randomized, placebo-controlled pilot trial suggest that combining dietary nitrate supplementation with RT shows promise for enhancing clinically relevant functional mobility in middle-aged adults. These functional improvements occurred despite equivalent gains in muscle strength and hypertrophy between groups, indicating that nitrate supplementation may modulate movement efficiency during self-selected walking conditions through mechanisms independent of muscular adaptation. Overall, these preliminary findings establish the feasibility of this combined intervention and provide a strong rationale for a future, larger-scale randomized controlled trial to definitively confirm these benefits and further investigate the underlying neural and physiological pathways.

## Data Availability

Raw data related to the current study outcomes will be provided upon reasonable request by emailing the corresponding author (jar0105@auburn.edu).

## Abbreviation

1RM: One Rep Max
5RM: Five Rep Mac
ANCOVA: Analyses of covariance
BMI: Body mass index
BRJ: Beet root juice
DXA: Dual-energy X-ray absorptiometry
FM/FFM: Fat mass to fat-free mass
CNS: Central nervous system
IMUs: Inertial measurement units
i6MWT: Instrumented 6-min Walk Test
iSAW: Instrumented Stand and Walk
iTUG: Instrumented Timed-Up and Go
NO: Nitric oxide
NO_3_^−^: Dietary nitrate
NOS: Nitric oxide synthase
PLA: Placebo
PRE: Pre-intervention
POST: Post-intervention
RIR: Repetitions in reserve
RT: Resistance training
VL: Vastus lateralis
